# Effects of U0126 and MK2206 on cell growth and re-growth of endometriotic stromal cells grown on substrates of varying stiffness

**DOI:** 10.1038/srep42939

**Published:** 2017-02-20

**Authors:** Sachiko Matsuzaki, Jean-Luc Pouly, Michel Canis

**Affiliations:** 1 CHU Clermont-Ferrand, CHU Estaing, Chirurgie Gynécologique, Clermont-Ferrand, France; 2 Clermont Université, Université d’Auvergne, ISIT UMR6284, Clermont-Ferrand, France; 3 CNRS, ISIT UMR6284, Clermont-Ferrand, France

## Abstract

Endometriosis is a common gynecological disorder responsible for infertility and pelvic pain. A complete cure for patients with endometriosis awaits new targets and strategies. Here we show that U0126 (a MEK inhibitor) and MK2206 (an AKT inhibitor) synergistically inhibit cell growth of deep endometriotic stromal cells (DES) grown on polyacrylamide gel substrates (PGS) of varying stiffness (2 or 30 kilopascal [kPa]) or plastic *in vitro*. No significant differences in cell proliferation were observed among DES, endometrial stromal cells of patients with endometriosis (EES) from the proliferative phase (P), EES-S (secretory phase) and EES-M (menstrual phase) compared to cells grown on a substrate of the same stiffness at both higher (U0126 [30 μM] and MK2206 [9 μM]) and lower (U0126 [15 μM] and MK2206 [4.5 μM]) combined doses. However, cell re-growth of DES after drug discontinuation was higher than that of EES-P and EES-S when cells were grown on rigid substrates at both combined doses. Combination U0126 and MK2206 treatment is more effective than each drug alone in cell growth inhibition of DES. However, further studies are required to investigate the mechanisms underlying high cell survival and proliferation after drug discontinuation for developing target therapies that prevent recurrence.

Endometriosis, a common gynecological disorder responsible for infertility and pelvic pain, is defined as the presence of endometrial glands and stroma within extra-uterine sites^1^. It affects approximately 10% of women of reproductive age^1^. However, despite extensive studies, its etiology, pathogenesis, and pathophysiology are not fully understood. A complete cure for patients with endometriosis awaits new targets and strategies.

We previously showed that the serine/threonine kinase AKT and extracellular regulated kinase (ERK) signaling pathways may cooperate to support growth of deep endometriotic lesions by enhancing endometriotic stromal cell proliferation and survival in a fibrotic microenvironment *in vitro*^2^. Our previous *in vitro* findings suggest that the AKT and ERK signaling pathways, both of which are important survival pathways, may compensate for each other, resulting in apoptosis resistance in endometriotic stromal cells^2^. Therefore, we speculated that cotargeting the PI3K/AKT/mTOR and RAF/MEK/ERK pathways may be effective for treatment of endometriosis.

Until now, the efficacy of numerous molecules has been evaluated in *in vitro* cell culture systems to develop novel strategies for treatment of endometriosis^3^,^4^. However, previous *in vitro* experiments have had at least two limitations. First, previous drug screening assays were performed in rigid plastic, which is much stiffer than that occurring *in vivo*. Studies have shown that matrix stiffness affects responsiveness to cytotoxic drugs in a cell-dependent and drug-dependent manner^5^,^6^,^7^. Our previous study showed that deep infiltrating endometriotic stromal cells (DES) can sense changes in extracellular matrix (ECM) stiffness and respond to them *in vitro*^8^. To investigate cell responses to drugs, it is critical to model *in vivo* tissue compliance conditions *in vitro*. Second, a high recurrence rate after medical treatment with or without surgery is a major clinical problem for patients with endometriosis^9^. However, to the best of our knowledge, no previous *in vitro* study evaluated whether candidate molecules for the treatment of endometriosis could prevent relapse of the disease after discontinuation of treatment. Before validation of the effects of candidate molecules can be performed in animal experiments or clinical trials, it is important to evaluate whether candidate molecules could decrease the number of cells that can survive treatment and consequently prevent re-growth of endometriotic cells *in vitro*.

The objective of the present study was to investigate whether combined treatment with U0126 and MK2206 can effectively inhibit cell proliferation during and after treatment in DES *in vitro*. We evaluated the effects of U0126 alone and MK2206 alone, as well as the combination of U0126 and MK2206, on inhibition of cell proliferation of DES, endometrial stromal cells with (EES), and/or without (NEES) endometriosis grown on polyacrylamide gel substrates (PGS) of varying stiffness (2 or 30 kilopascal [kPa]) or plastic. In addition, we evaluated proliferation of viable cells after discontinuation of combined treatment with U0126 and MK2206 in DES, EES, and/or NEES grown on PGS of varying stiffness (2 or 30 kPa) or plastic. For any disease, ideal drugs are those that increasing the probability of the disease cure and decrease normal tissue toxicity. In addition, studies have shown that endometrium of patients with endometriosis may differ biochemically from that of patients without endometriosis. Our previous study showed that levels of phosphorylated AKT and phosphorylated ERK were significantly higher in menstrual endometrium *in vivo* and *in vitro* in patients with endometriosis compared to those of patients without endometriosis^2^. Therefore, in the present study, both EES and NEES were included for comparison. In the present study, we elected to use PGS of two different degrees of stiffness, 2 (soft) or 30 (rigid) kPa, based on the results of our previous study^8^. The soft substrate (2-kPa PGS) and the rigid substrate (30-kPa PGS) may mimic *in vivo* tissue compliance of the endometrium or deep infiltrating endometriosis (DIE), respectively^8^.

## Results

### Drug combination analysis

Combined treatment with U0126 and MK2206 produced a synergic effect in DES grown on substrates of varying stiffness (2- or 30-kPa PGS, or plastic) for ED 95 (effect dose at which 95% growth inhibition occurs), ED 90, and ED 75 (See Supplementary Fig. S1 & Supplementary Table S1). For ED 50, when DES were grown on 30-kPa PGS or plastic, an additive or an antagonistic effect was produced, whereas in cells grown on 2-kPa PGS, a synergic effect was observed (See Supplementary Fig. S1 & Supplementary Table S1). In EES derived from the proliferative phase (EES-P), EES derived from the secretory phase (EES-S) and EES derived from the menstrual phase (EES-M), combined U0126 and MK2206 treatment produced an additive or antagonistic effect in cells grown on substrates of varying stiffness (2- or 30-kPa PGS, or plastic) (See Supplementary Fig. S1 & Supplementary Table S1).

### Effects of the combination of U0126 and MK2206 on inhibition of cell proliferation of DES, EES, and NEES

#### DES versus EES

**Figure 1:**
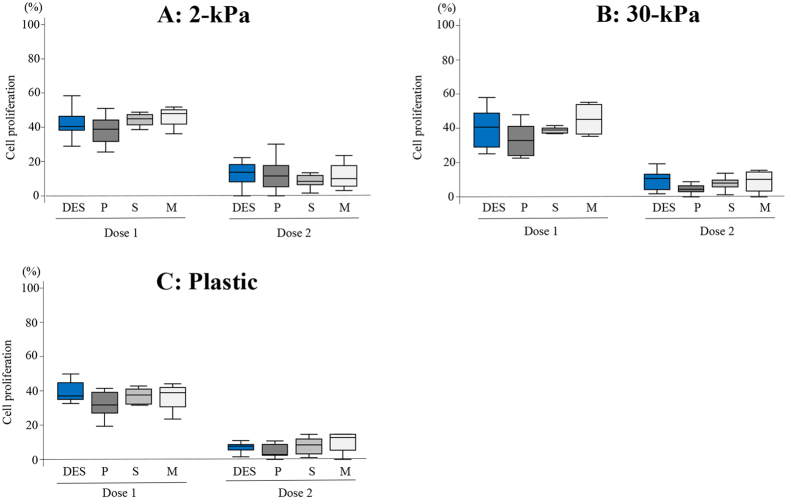
Comparison of cell proliferation of deep endometriotic stromal cells DES (n = 14), endometrial stromal cells of patients with endometriosis (EES) derived from the proliferative phase (EES-P) (n = 10), EES derived from the secretory phase (EES-S) (n = 6) and EES derived from the menstrual phase (EES-M) (n = 5) grown on PGS of varying stiffness (2 (**A**) or 30 kPa (**B**)) or plastic (**C**) treated with combination U0126 and MK2206. Dose 1: U0126 (15 μM) and MK2206 (4.5 μM). Dose 2: U0126 (30 μM) and MK2206 (9 μM). P: EES-P. S: EES-S. M: EES-M. Numerical values are presented as box and whisker plots showing medians and the smallest and largest data points ≤1.5 × IQR from the 25th and 75th percentiles, respectively.

No significant differences in cell proliferation were observed among DES, EES-P, EES-S, and EES-M compared to cells grown on a substrate of the same stiffness (2- or 30-kPa PGS, or plastic) at both higher (U0126 [30 μM] and MK2206 [9 μM]) and lower (U0126 [15 μM] and MK2206 [4.5 μM]) combined doses (Fig. 1).

#### EES versus NEES

No significant differences in cell proliferation were observed between EES and NEES (EES-P versus NEES-P, EES-S versus NEES-S, or EES-M versus NEES-M) when compared to cells grown on a substrate of the same stiffness (2- or 30-kPa PGS, or plastic) at both higher (U0126 [30 μM] and MK2206 [9 μM]) and lower (U0126 [15 μM] and MK2206 [4.5 μM]) combined doses (See Supplementary Fig. S2).

#### Effects of substrates of varying stiffness (2- or 30-kPa PGS, or plastic) on inhibition of cell proliferation

**Figure 2:**
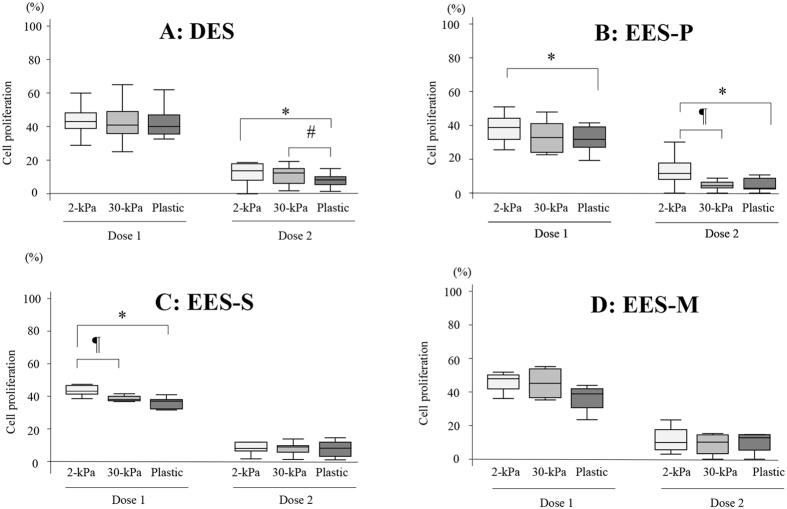
Effects of combined treatment with U0126 and MK2206 on cell proliferation in DES (**A**) (n = 14), EES-P (**B**) (n = 10), EES-S (**C**) (n = 6), or EES-M (**D**) (n = 5). Cells were grown on PGS of varying stiffness (2 or 30 kPa) or plastic. Dose 1: U0126 (15 μM) and MK2206 (4.5 μM). Dose 2: U0126 (30 μM) and MK2206 (9 μM). Numerical values are presented as box and whisker plots showing medians and the smallest and largest data points ≤1.5 × IQR from the 25th and 75th percentiles, respectively. *p < 0.05: 2-kPa PGS versus plastic. ^#^p < 0.05: 30-kPa PGS versus plastic. ^¶^p < 0.05: 2-kPa versus 30-kPa PGS.

In DES (Fig. 2), cell proliferation was significantly more inhibited in cells grown on plastic than those grown on 2-kPa or 30-kPa PGS, when cells were treated with a higher (U0126 [30 μM] and MK2206 [9 μM]) combined dose. However, no significant effects of substrates of varying stiffness (2- or 30-kPa PGS, or plastic) on cell proliferation of DES were observed when cells were treated with a lower (U0126 [15 μM] and MK2206 [4.5 μM]) combined dose (Fig. 2). In EES-P, EES-S, NEES-P and NEES-S, cell proliferation was significantly more inhibited in cells grown on plastic or 30-kPa PGS compared to those grown on 2-kPa PGS when cells were treated with a higher combined dose (U0126 [30 μM] and MK2206 [9 μM]) and/or a lower combined dose (U0126 [15 μM] and MK2206 [4.5 μM]) (See Supplementary Fig. S3). No significant effect of substrates of varying stiffness (2- or 30-kPa PGS, or plastic) was observed on cell proliferation of either EES-M or NEES-M (See Supplementary Fig. S3) treated with either a higher (U0126 [30 μM] and MK2206 [9 μM]) or lower (U0126 [15 μM] and MK2206 [4.5 μM]) combined dose.

### Effects of treatment with either U0126 alone, MK2206 alone, or the combination of U0126 and MK2206 on apoptosis

**Figure 3:**
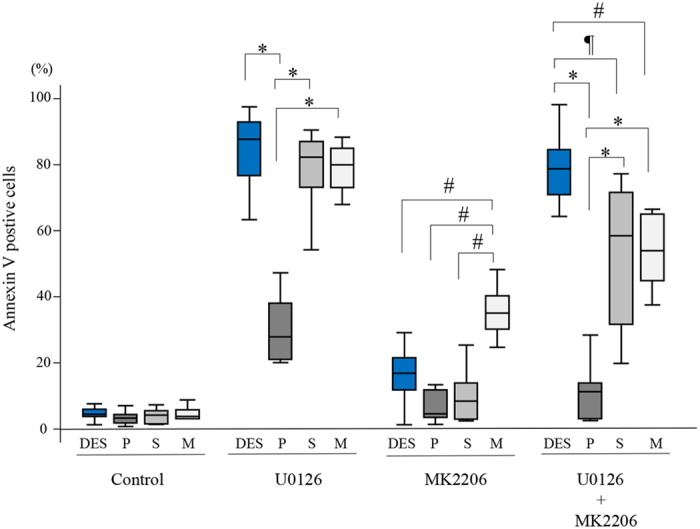
Effects of either U0126 (30 μM) alone, MK2206 (9 μM) alone, or combination U0126 (30 μM) and MK2206 (9 μM) on Annexin V-positive cells of DES (n = 12), EES-P (n = 6), EES-S (n = 6), and EES-M (n = 5). Cells were grown on PGS of varying stiffness (2 or 30 kPa) or plastic. P: EES-P. S: EES-S. M: EES-M. Numerical values are presented as box and whisker plots showing medians and the smallest and largest data points ≤1.5 × IQR from the 25th and 75th percentiles, respectively. *p < 0.05 versus EES-P. ^¶^p < 0.05 versus EES-S. ^#^p < 0.05 versus EES-M.

The percentage of Annexin V-positive cells treated with U0126 alone was significantly higher in DES, EES-S, and EES-M compared to that in EES-P (Fig. 3). When cells were treated with MK2206 alone, the percentage of Annexin V-positive cells was significantly higher in EES-M compared to that in DES, EES-P, and EES-S (Fig. 3). When cells were treated with combination U0126 and MK2206, the percentage of Annexin V-positive cells was significantly higher in DES compared to that in EES-P, EES-S, and EES-M (Fig. 3). In addition, the percentage of Annexin V-positive cells was significantly higher in EES-S and EES-M compared to that in EES-P treated with combination U0126 and MK2206 (Fig. 3).

### Effects of treatment with either U0126 alone, MK2206 alone, or the combination of U0126 and MK2206 on markers of cellular senescence

**Figure 4:**
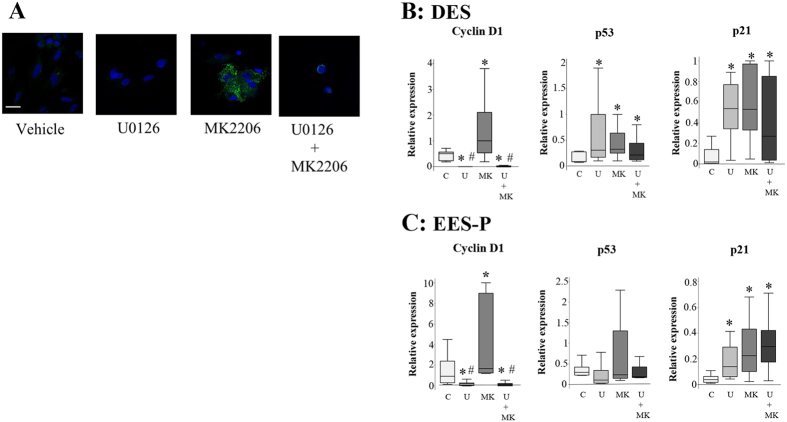
(**A**) Representative photomicrograph of cytochemical staining of senescence-associated beta-galactosidase (SA-βgal) activity in DES. SA-βgal activity in DES treated with either vehicle (DMSO) alone, U0126 (30 μM) alone, MK2206 (9 μM) alone, or combination U0126 (30 μM) and MK2206 (9 μM). Scale bar: 50 μm. (**B**,**C**) Effects of either U0126 (30 μM) alone, MK2206 (9 μM) alone, or combination U0126 (30 μM) and MK2206 (9 μM) on mRNA levels of cyclin D1, p53, and p21 in DES (**B**) and EES-P (**C**) from the same patients (n = 6). *p < 0.05 versus control. ^#^p < 0.05 versus MK2206. Numerical values are presented as box and whisker plots showing medians and the smallest and largest data points ≤1.5 × IQR from the 25th and 75th percentiles, respectively. Levels of cyclin D1, p53, and p21 mRNAs are presented relative to the level of the reference gene, GAPDH. C: control, U: U0126, MK: MK2206, U + MK: U0126 + MK2206.

SA-βgal activity was observed in DES and EES-P treated with MK2206 alone (Fig. 4A). Levels of cyclin D1 mRNA were significantly higher in both DES and EES-P treated with MK2206 alone compared to the vehicle-treated control (Fig. 4B,C). Levels of p53 and p21 mRNAs of DES and those of p21 mRNA in EES-P were significantly higher in cells treated with U0126 alone, MK2206 alone, or combination U0126 and MK2206 compared to cells treated with vehicle alone (Fig. 4B,C).

### Cell proliferation after a 72-h discontinuation of combination U0126 and MK2206

#### DES versus EES

**Figure 5:**
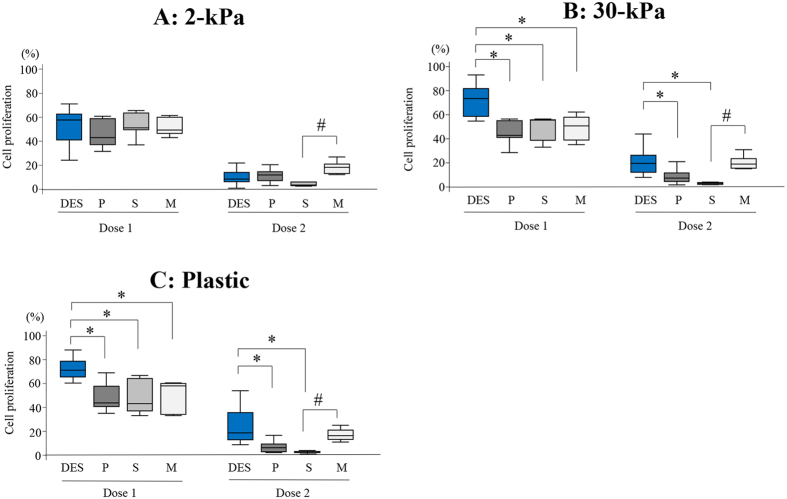
Comparison of cell proliferation of DES (n = 14), EES-P (n = 10), EES-S (n = 6) and EES-M (n = 5) grown on PGS of varying stiffness (2 (**A**) or 30 kPa (**B**)) or plastic (**C**) after a 72-h discontinuation of U0126 and MK2206. P: EES-P. S: EES-S. M: EES-M. Dose 1: U0126 (15 μM) and MK2206 (4.5 μM). Dose 2: U0126 (30 μM) and MK2206 (9 μM). *p < 0.05 versus DES. ^#^p < 0.05 versus EES-M. Numerical values are presented as box and whisker plots showing medians and the smallest and largest data points ≤1.5 × IQR from the 25th and 75th percentiles, respectively.

When cells were grown on 2-kPa PGS, cell proliferation of EES-M after a 72-h drug discontinuation was significantly higher than that of EES-S at a higher (U0126 [30 μM] and MK2206 [9 μM]) combined dose (Fig. 5). When cells were grown on 30-kPa PGS or plastic, cell proliferation after a 72-h drug discontinuation was significantly higher in DES compared to that of EES-P, EES-S, and EES-M at a lower (U0126 [15 μM] and MK2206 [4.5 μM]) combined dose and compared to that of EES-P and EES-S at a higher (U0126 [30 μM] and MK2206 [9 μM]) combined dose (Fig. 5). In addition, cell proliferation of EES-M grown on 30-kPa PGS or plastic was significantly higher than that of EES-S after a 72-h drug discontinuation of a higher (U0126 [30 μM] and MK2206 [9 μM]) combined dose (Fig. 5).

#### EES versus NEES

When a lower (U0126 [15 μM] and MK2206 [4.5 μM]) combined dose was applied, no significant difference in cell proliferation was observed after a 72-h drug discontinuation between EES and NEES (EES-P versus NEES-P, EES-S versus NEES-S, or EES-M versus NEES-M) compared to cells grown on a substrate of the same stiffness (2- or 30-kPa PGS, or plastic) (See Supplementary Fig. S4). However, when a higher (U0126 [30 μM] and MK2206 [9 μM]) combined dose was applied, cell proliferation after a 72-h drug discontinuation was significantly higher in EES-P than in NEES-P when cells were grown on 2-kPa PGS, and significantly higher in EES-M than in NEES-M when compared to cells grown on a substrate of the same stiffness (2- or 30-kPa, or plastic) (See Supplementary Fig. S4). No significant difference in cell proliferation after a 72-h drug discontinuation was observed between EES-S and NEES-S when compared to cells grown on a substrate of the same stiffness (2- or 30-kPa PGS, or plastic) (See Supplementary Fig. S4).

#### Effects of substrates of varying stiffness (2- or 30-kPa PGS, or plastic) on cell survival

**Figure 6:**
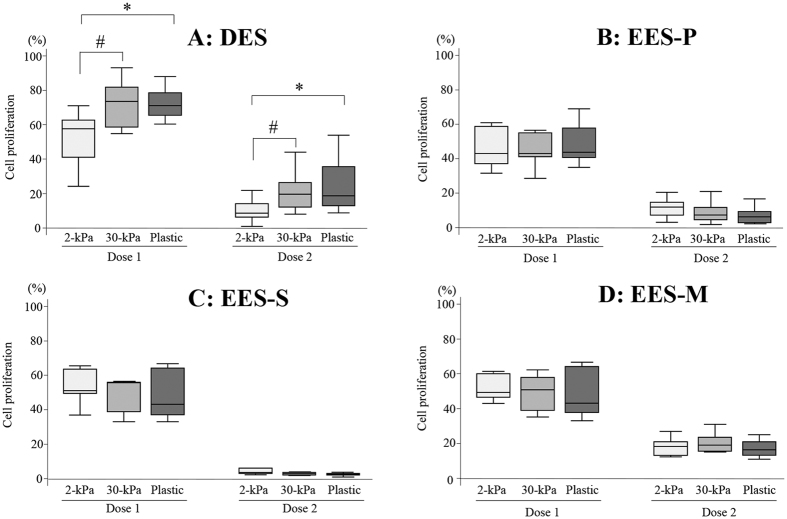
Cell proliferation in DES (**A**) (n = 14), EES-P (**B**) (n = 10), EES-S (**C**) (n = 6), or EES-M (**D**) (n = 5) after a 72-h drug discontinuation of combination U0126 and MK2206. Cells were grown on PGS of varying stiffness (2- or 30-kPa PGS) or plastic. Dose 1: U0126 (15 μM) and MK2206 (4.5 μM). Dose 2: U0126 (30 μM) and MK2206 (9 μM). *p < 0.05: 2-kPa PGS versus plastic. ^#^p < 0.05: 2-kPa versus 30-kPa PGS.

Cell proliferation of DES was significantly higher in cells grown on 30-kPa PGS and plastic than those grown on 2-kPa PGS after a 72-h drug discontinuation at either a higher (U0126 [30 μM] and MK2206 [9 μM]) or lower (U0126 [15 μM] and MK2206 [4.5 μM]) combined dose (Fig. 6). No significant differences in cell proliferation of either EES (-P, -S, or -M) or NEES (-P, -S, or -M) (See Supplementary Fig. S5) grown on substrates of varying stiffness (2- or 30-kPa PGS, or plastic) were observed after a 72-h drug discontinuation at either a higher (U0126 [30 μM] and MK2206 [9 μM]) or lower (U0126 [15 μM] and MK2206 [4.5 μM]) combined dose.

#### Intra-group comparisons

Cell proliferation after a 72-h drug discontinuation was significantly increased compared to that after a 48-h treatment in DES, EES (EES-P, EES-S and EES-M) and NEES (NEES-P, NEES-S and NEES-M) grown on substrates of varying stiffness (2- or 30-kPa PGS, or plastic) at a lower combined dose (See Supplementary Fig. S6). At a higher combined dose, cell proliferation after a 72-h drug discontinuation was significantly increased in DES grown on rigid substrates (30-kPa PGS or plastic), and EES-M grown on substrates of varying stiffness (2- or 30-kPa PGS, or plastic) (See Supplementary Fig. S6).

### Effects of an autophagy inhibitor on cell survival of DES after a 72-h discontinuation of combination U0126 and MK2206

**Figure 7:**
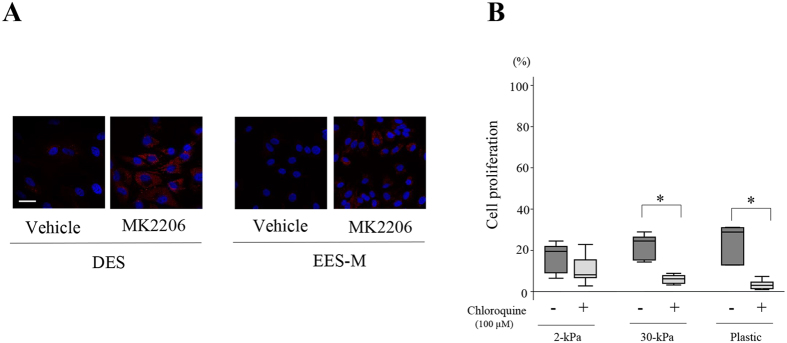
(**A**) Representative photomicrograph of LC3A/B expression in DES and EES-M. LC3A/B expression in DES and EES-M treated with either vehicle (DMSO) alone or MK2206 (9 μM) alone. Scale bar: 50 μm. (**B**) Effects of the combined treatment of U0126 and MK2206 with versus without chloroquine on cell proliferation of DES (n = 6) after a 72-h drug discontinuation. Cells were grown on PGS of varying stiffness (2 or 30 kPa) or plastic. *p < 0.05 with versus without chloroquine. Numerical values are presented as box and whisker plots showing medians and the smallest and largest data points ≤1.5 × IQR from the 25th and 75th percentiles, respectively.

LC3-positive puncta, a marker for autophagy, were observed in DES, EES (-P, -S, and-M), and NEES (-P, -S, and-M) treated with MK2206 (Fig. 7A). Cell proliferation of DES after a 72-h discontinuation of combination U0126 (30 μM) and MK2206 (9 μM) with chloroquine (100 μM) was significantly lower than that without chloroquine when DES were grown on rigid substrates (30-kPa PGS or plastic) (Fig. 7B).

## Discussion

The present study showed a synergic effect of combined treatment with U0126 and MK2206 on DES, whereas an additive or antagonistic effect was observed on EES. The present study supports our speculation that cotargeting the PI3K/AKT/mTOR and RAF/MEK/ERK pathways may be effective for treatment of endometriosis^2^.

In the present study, we observed that the inhibition of cell proliferation was significantly higher in DES grown on plastic than those grown on 2- or 30-kPa PGS. One study evaluated a total of 18 small-molecule known or suspected inhibitors of cell proliferation in lung fibroblasts grown on soft (1-kPa PGS) or rigid (glass) substrates^5^. The study investigators identified compounds with both increased and decreased potency on soft relative to rigid substrates, in addition to those with equivalent efficacy irrespective of substrate stiffness^5^. These findings and our present findings suggest that when drug screening assays are performed in rigid plastic/glass, drug efficacy may be under- or over-estimated.

Akt is well known for its anti-apoptotic activity^9^. However, the level of apoptosis as evaluated by Annexin V-positive cells did not appear to be sufficiently explained for the inhibition of cell proliferation in cells treated with MK2206 alone. Studies showed that Akt knockdown or inactivation with small-molecule inhibitors did not induce significant apoptosis^10^,^11^. Another mechanism may be responsible for inhibition of cell proliferation by MK2206. Our previous western blot analysis showed that MK2206 alone significantly increased levels of phosphorylated ERK in DES compared to those of vehicle-treated cells^2^. A hyperactivated ERK-driven transcriptional induction of the cyclin-dependent kinase inhibitor p21 and cyclin D1 triggers a massive accumulation of both cyclin D1 and p21, leading to cell cycle arrest by p21^12^. p21 mediates the tumor suppressor p53-dependent G1 growth arres^13^. When the cell cycle is blocked, while growth-promoting pathways remain active, cells continue to grow in size and undergo cellular senescence^12^. Cyclin D1 is the driving force of cell cycle transition from G1 to S phase in proliferating cells^14^. However, paradoxically, senescent cells have much higher levels of cyclin D1 than proliferating cells^15^,^16^. The present study showed the presence of beta-galactosidase activity, and increased cyclin D1 and p21 mRNA levels, which are biomarkers for cellular senescence^17^, in MK2206-treated cells. We speculated that abnormal hyperactivation of ERK through MK2206 may promote cellular senescence and result in inhibition of proliferation of MK2206-treated cells.

The present results showed that cell proliferation after discontinuation of the combined treatment was significantly higher in DES grown on 30-kPa PGS or plastic than those grown on 2-kPa PGS. However, we observed no significant effects of varying stiffness (2- or 30-kPa PGS, or plastic) on cell proliferation of either EES or NEES after drug discontinuation. These findings suggest that DES grown on a rigid substrate may have more potential to relapse than those grown on a soft substrate. Our findings appear to agree with the clinical evidence of a high recurrence rate following medical treatment in endometriosis^18^. It may be necessary to interrupt mechanical interactions between endometriotic cells and their surrounding ECM to prevent recurrence after medical treatment. The present *in vitro* findings may not support the future clinical use of the combined treatment with U0126 and MK2206 in patients with DIE, because of high cell survival and proliferation after drug discontinuation.

In the present study, we further attempted to investigate a potential mechanism underlying cell survival in DES treated with combination U0126 and MK2206. We observed a significantly higher percentage of Annexin V-positive cells in DES than in EES-P, -S, and -M when treated with combination U0126 and MK2206. Nevertheless, we observed higher proliferation of DES after drug discontinuation than of EES-P and EES-S when cells were grown on rigid substrates (30-kPa PGS or plastic). Studies showed that Akt knockdown or inactivation with small-molecule inhibitors markedly increased autophagy^17^,^19^,^20^,^21^. Autophagy is a highly conserved process in eukaryotes in which organelles, proteins, or lipids are sequestered into double-membrane vesicles termed autophagosomes for degradation and eventual recycling^22^. Inhibiting autophagy can either promote or inhibit cell death depending on the conditions and agents used^23^. Previous studies showed that MK2206 treatment induced autophagy in various cells types, and suppression of autophagy enhances cell death in an intracranial glioma mouse model^19^ and in melanoma cells^21^, whereas it inhibits cell death in PTEN-mutant gastric cancer cells^20^. A recent study demonstrated upregulation of autophagy in ovarian endometriosis^24^. In addition, a recent study showed that hydroxychloroquine, an autophagy inhibitor, could decrease lesion numbers and disrupt lesion histopathology in a mouse model of endometriosis^25^. The present histochemical analysis revealed the presence of LC3-positive puncta in MK2206-treated DES^26^. In addition, we observed significantly lower proliferation of DES after discontinuation of treatment with U0126, MK2206, and chloroquine than with U0126 and MK2206 when cells were grown on rigid substrates. The present findings suggest that MK2206 treatment may induce autophagy, which may inhibit cell death, resulting in cell survival from combined treatment with U0126 and MK2206 and subsequent cell proliferation. However, the present analysis has limitations. The appearance of LC3-positive puncta does not necessarily indicate high levels of active autophagy^26^. In addition, most currently available chemical inhibitors of autophagy, including chloroquine, hydroxychloroquine, and bafilomycin A1, are not entirely specific^27^. Further studies are required to determine whether autophagy is involved in the high relapse rate of endometriosis after medical treatment.

Another potential explanation is that stem-like cells in endometriosis and menstrual endometrium of patients with endometriosis may be responsible for high cell survival and proliferation after discontinuation of combined treatment with U0126 and MK2206. A growing body of evidence suggests that endometriosis may arise from stem cells^28^. It has been proposed that endometrial stem/progenitor cells with associated niche cells are abnormally shed during menses, which may then implant into the peritoneal cavity by retrograde menstruation^28^. Endometriosis is a benign disease. However, studies have shown that endometriosis shares many aspects with cancer. It has been proposed that small subsets of cancer cells with extremely high tumorigenic potential, termed cancer stem cells (CSCs) or stem-like cancer cells, are responsible for relapse after cancer treatments such as chemotherapy or radiotherapy^29^,^30^. A recent study demonstrated that inhibition of cancer stemness effectively suppressed relapse and metastasis in a pancreatic cancer xenograft model^31^. In addition, preclinical data suggest that autophagy plays a crucial role in the origin, maintenance, and systemic distribution of CSCs^32^. Recent studies showed that pharmacologically altering CSC-related autophagy can overcome CSC resistance^33^,^34^. These findings led us to speculate that autophagy in stem-like cells in endometriosis may play a role in recurrence after medical treatment. Further studies are required to characterize DES and EES-M that can survive combined treatment with U0126 and MK2206 and subsequently proliferate after discontinuation of the combined treatment. Such investigations would provide further information for developing target therapies that prevent or minimize recurrence after medical treatment for endometriosis.

The present *in vitro* model has many limitations: endometriotic tissue and endometrium are composed of multiple cell types and extracellular matrix, but in the present study, only endometriotic and endometrial stromal cells were cultured based on our previous findings. Second, endometriotic tissue and endometrium are three-dimensional (3D), but the present studies used a conventional two-dimensional (2D) culture system. 3D *in vitro* models have been considered to span the gap between 2D cell cultures and whole-animal systems^35^,^36^. Further efforts are required to develop better culture systems that mimic the cellular complexity typical of *in vivo* endometriotic tissues.

In conclusion, the present study showed that combined treatment with U0126 and MK2206 synergistically inhibited cell proliferation of DES. However, cell proliferation of DES after drug discontinuation was higher than that of EES-P and EES-S when cells were grown on rigid substrates. The present *in vitro* findings may not support the future clinical use of the combined treatment with U0126 and MK2206 in patients with DIE. Further studies are required to investigate the mechanisms underlying high cell survival and proliferation after drug discontinuation for developing target therapies that prevent recurrence.

## Materials and Methods

### Patients

Patients aged 20–37 years undergoing laparoscopy for endometriosis were recruited at CHU Clermont-Ferrand, France. None of the women had received hormonal therapy and none used intrauterine contraception for at least 6 months prior to surgery. Recruited patients had regular menstrual cycles (26–32 days) with confirmation of their menstrual history. Endometrial and endometriotic samples from 73 patients who had histological evidence of rectovaginal DIE were used for the present analysis. In addition, endometrial tissues from 21 patients without endometriosis were obtained. The clinical characteristics of patients are shown in Supplementary Table S2. The research protocol was approved by the Consultative Committee for Protection of Persons in Biomedical Research (CPP) of the Auvergne (France) region. All experiments were performed in accordance with the approved guidelines and regulations. Informed written consent was obtained from each patient prior to tissue collection.

### Cell culture

DES, EES, and NEES were isolated as previously described (Supplementary methods)^2^,^8^,^37^,^38^,^39^. Cells at passage 1 were used for experiments. The numbers of samples of DES, EES, and/or NEES used for each experiment are summarized in Supplementary Table S3. Immunofluorescence staining was performed to determine the purity of the isolated EES, NEES and DES as previously described^2^,^8^,^37^,^38^,^39^.

### Preparation of stiffness-controlled 96-well plates

Stiffness-controlled 96-well plates were prepared using modifications to the protocol of Syed *et al*.^40^ (See Supplementary Methods).

### Cell proliferation assays and drug combination analysis

Cell proliferation assays were performed using the CellTiter 96^®^ AQueous One Solution Cell Proliferation Assay (MTS) (Promega, Charbonnières-les-Bains, France), as previously described^37^,^38^,^39^. Briefly, cells (5 × 10^3^ cells per well) from the same samples were plated on 2- or 30-kPa PGS or plastic in triplicate in 96-well plates. After 2 h at 37 °C and 5% CO_2_ to allow cell adhesion and spreading, drugs were added at the indicated concentration with 100 μL culture media (2% charcoal-stripped FBS), individually or in combinations. U0126 (Selleck Chemicals, Houston, TX, USA) or MK2206 (Selleck Chemicals) were dissolved in dimethyl sulfoxide (DMSO) (Life Technologies). Chloroquine (Sigma-Aldrich) was dissolved in phenol red-free DMEM/F-12. The Chou-Talalay model calls for cytotoxic agents to be used at a fixed dose ratio^41^, so we elected to use U0126 and MK2206 in a 10:3 molar ratio based on the results of our previous study^2^. To calculate the combination index (CI) after 48 h of treatment, we used five different doses of U0126 and MK2206. To evaluate the effects of the combination of U0126 and MK2206 on inhibition of cell proliferation and cell survival after drug discontinuation, cells from the same samples were divided into two: one set was used to evaluate inhibition of cell proliferation after a 48-h treatment and the other set was used to evaluate cell proliferation of viable cells 72 h after drug discontinuation. We used two different doses of U0126 and MK2206 based on the results of prior experiments for CI. To evaluate inhibition of cell proliferation after the 48-h treatments, 20 μL of MTS were added to all wells and incubated for 2 h at 37 °C. To evaluate cell proliferation of viable cells after the 72-h drug discontinuations, cells were washed twice with PBS after a 48-h treatment, followed by a 72-h culture in drug-free medium with 10% FBS. Then, 20 μL of MTS were added to all wells and incubated for 2 h at 37 °C. Prior to absorbance measurements, 80 μL of the MTS:medium solution were transferred from each well into a well of a new 96-well plate to avoid background absorbance from the gels. Absorbance in the no-gel 96-well plate was measured at 490 nm (Spectra Max Plus, Molecular Devices, Sunnyvale, CA, USA). Percent cell proliferation was calculated as percent of vehicle control. CalcuSyn software (Biosoft, Great Shelford, Cambridge, UK) was used to calculate the CI according to the median-effect method of Chou and Talalay^42^,^43^. CI values <0.9, 0.9–1.1, and >1.1 represent synergism, additivity, and antagonism, respectively.

### Analysis of apoptosis by flow cytometry

Cells (1 × 10^6^ cells) were seeded onto Primaria flasks (BD Biosciences). After 2 h at 37 °C and 5% CO_2_ to allow for cell adhesion and spreading, cells were incubated with culture media (2% charcoal-stripped FBS) containing either U0126 alone (30 μM) (Sigma-Aldrich), MK2206 alone (9 μM) (Sigma-Aldrich), a combination of U0126 (30 μM) and MK2206 (9 μM), or vehicle (DMSO) for 24 h. Cells were stained with Annexin V-FITC and PI (Annexin V kit, Beckman Coulter, Villepinte, France) and evaluated for apoptosis by flow cytometry analyses using a BD LSRII flow cytometer (BD Biosciences) according to the manufacturer’s protocol. Both early apoptotic (Annexin V-positive, PI-negative) and late (Annexin V-positive and PI-positive) apoptotic cells were included in cell death determinations.

### Immunofluorescence staining for light chain 3 isoforms A and B (LC3A/B) proteins and senescence-associated beta-galactosidase (SA-βgal) activity

Immunofluorescence staining for LC3A/B (D2H10, 1:100, Cell Signaling, Danvers, MA, USA) was performed. Fluorescence histochemical detection of SA-βgal activity was performed according to the protocol published by Debacq-Chainiaux *et al*.^44^. (See Supplementary Methods).

### RNA extraction, RNA yield and integrity, and quantitative real-time RT-PCR

Cells were seeded onto 24-well plates (5 × 10^4^ cells per well). After 2 h at 37 °C and 5% CO_2_ to allow for cell adhesion and spreading, cells were incubated with culture media (2% charcoal-stripped FBS) containing either U0126 (30 μM) (Sigma-Aldrich), MK2206 (9 μM) (Sigma-Aldrich), U0126 (30 μM) and MK2206 (9 μM), or vehicle (DMSO) only for 24 h. Total RNA was extracted using the Qiagen RNeasy Mini Kit according to the manufacturer’s instructions (Qiagen, Courtaboef, France) as previously described^2^,^8^,^37^,^38^,^39^. RNA yield and integrity were analyzed using the RNA 6000 Pico kit and the Agilent Bioanalyzer 2100 (Agilent Technologies, Santa Clara, CA, USA) as previously described^2^,^8^,^37^,^38^,^39^. mRNA levels of cyclin D1, p53, and p21^WAF1/Cip1^ (p21) were measured by quantitative real-time RT-PCR with a Light Cycler (Roche, Mannheim, Germany) as previously described^2^,^8^,^37^,^38^,^39^. Here, Primer sets are shown in Supplementary Table S4.

### Statistical analysis

The STATA program version 12 (StataCorp, College Station, TX, USA) was used for statistical analysis. Comparisons between different groups were made using one-way analysis of variance following Scheffé’s method, the Mann-Whitney *U* test, or the Wilcoxon matched pairs signed-ranks test. Statistical significance was defined as p < 0.05.

## Additional Information

**How to cite this article**: Matsuzaki, S. *et al*. Effects of U0126 and MK2206 on cell growth and re-growth of endometriotic stromal cells grown on substrates of varying stiffness. *Sci. Rep.* **7**, 42939; doi: 10.1038/srep42939 (2017).

**Publisher's note:** Springer Nature remains neutral with regard to jurisdictional claims in published maps and institutional affiliations.

## Supplementary Material

Supplementary Information
