# Supplementary material for: Effects of U0126 and MK2206 on cell growth and re-growth of endometriotic stromal cells grown on substrates of varying stiffness
Source: Sci Rep. 2017 Feb 20;7:42939. doi: 10.1038/srep42939 (PMC5317159; doi:10.1038/srep42939)
Supplement: Supplementary Information [file srep42939-s1.pdf]

Effects of U0126 and MK2206 on cell growth and re-growth of endometriotic stromal cells grown on substrates of varying stiffness

Sachiko Matsuzaki [1, 2, 3 *]

Jean-Luc Pouly [1]

Michel Canis [1, 2, 3]

[1] CHU Clermont-Ferrand, CHU Estaing, Chirurgie Gynécologique, Clermont-Ferrand, France

[2] Clermont Université, Université d'Auvergne, ISIT UMR6284, Clermont-Ferrand, France

[3] CNRS, ISIT UMR6284, Clermont-Ferrand, France

***Corresponding Author: Sachiko Matsuzaki**

CHU Clermont-Ferrand, CHU Estaing, Chirurgie Gynécologique, 1, Place Lucie et Raymond Aubrac, 63003 Clermont-Ferrand, France, Tel: +33 4 73 75 01 38, E-mail: sachikoma@aol.com

Supplementary Methods

Cell culture

DES, EES, and NEES were isolated as previously described.[1-5] The endometrial and endometriotic tissues were carefully dissected and minced into 1–2 mm3 fragments incubated in phenol red-free DMEM/F-12 containing type I collagenase (0.25%) (Life Technologies) and deoxynuclease I (15 U/mL) (Life Technologies) for 60 min (endometrium) or 90 min (endometriosis) at 37°C. Endometrial or endometriotic cells were then separated by filtration through a 40-µm nylon cell strainer (BD, Le Pont de Claix, France). Epithelial cells that remained intact were retained by the strainer, whereas dispersed stromal cells passed through the strainer into the filtrate. Red blood cells were removed by hypotonic lysis (NH4Cl, 0.15 mol/L; KHCO3, 1 mmol/L; Na2 EDTA, 0.1 mmol/L) (Life Technologies). Isolated cells were plated onto Primaria flasks (BD Biosciences, Le Pont-De-Claix, France) in phenol red-free Dulbecco's modified Eagle's medium (DMEM)/F-12 (Life Technologies, Cergy Pontoise, France) containing 10% charcoal-stripped fetal bovine serum (FBS) (Sigma-Aldrich, Lyon, France), 100 U/mL penicillin (Sigma-Aldrich), 0.1 mg/mL streptomycin (Sigma-Aldrich), and 0.25 µg/mL amphotericin B (Sigma-Aldrich) and incubated at 37 °C in 95% air/5% CO2. Cells at passage 1 were used for experiments. The numbers of samples of DES, EES, and/or NEES used for each experiment are summarized in Supplementary Table S3 online.

Immunofluorescence staining was performed to determine the purity of the isolated EES, NEES and DES using monoclonal antibodies for human cytokeratin (a marker of epithelial cells) (MNF116, 1:100, DAKO, Glostrup, Denmark), vimentin (a marker of stromal cells) (V9, 1:100, DAKO), CD10 (a marker of stromal cells) (F-4, 1:25, Santa Cruz Biotechnology, Santa Cruz, CA, USA), smoothelin (a marker of smooth muscle cells) (MAB3242, 1:200, Merck Millipore, Molsheim, France),[6,7] factor VIII (a marker of endothelial cells) (1:100, DAKO), and CD45 (a marker of bone marrow-derived cells) (1:100, DAKO), as previously described.[1-5] The results indicated that the purity of stromal cells was >99% (data not shown).

Preparation of stiffness-controlled 96-well plates

Stiffness-controlled 96-well plates were prepared using modifications to the protocol of Syed et al.[8] Briefly, to produce the desired gels of variable stiffness (2 or 30 kPa), polyacrylamide gel precursor solution was prepared by mixing acrylamide (Bio-Rad Laboratories, Hercules, CA, USA), bis-acrylamide (Bio-Rad), 1 M HEPES (Sigma-Aldrich), and double-distilled H_2O. The gel solution was pipetted onto the hydrophilic side of the flexible plastic support (GelBond PAG Gel Film, GE Healthcare Buckinghamshire, UK) and sandwiched with hydrophobic-coated glass slides. The polyacrylamide gel was then polymerized and dried onto the flexible plastic support. Then, the polyacrylamide gels were cut using a heavy-duty hole punch with a diameter of 6 mm for a 96-well plate. To glue the gels to the bottom of the 96-well plates, 5 μL of polydimethylsiloxane (PDMS, Sigma-Aldrich) were placed in the center of each well. Then, one polyacrylamide gel was placed in each well. To allow the PDMS to cure, the assembled 96-well plate was left at 37 °C for 4 h. Then, the polyacrylamide gel was activated with sulfo-SANPAH (Thermo Scientific, Illkirch, France) to cross-link the ECM protein (collagen) to the polyacrylamide gel surface. The polyacrylamide gel was coated with type I collagen (0.2 mg/mL) (BD Biosciences) overnight at 4 °C. The gels were sterilized under UV light (λ=200 nm) for 2 h. Then, the gels were soaked in complete medium overnight to hydrate and equilibrate.

Immunofluorescence staining for light chain 3 isoforms A and B (LC3A/B) proteins and senescence-associated beta-galactosidase (SA-βgal) activity

Cells were seeded onto glass cover slips (22 mm x 22 mm) (5×10^4 cells per cover slip). After 2 h at 37 °C and 5% CO_2 to allow for cell adhesion and spreading, cells were incubated with 2 mL culture media (2% charcoal-stripped FBS) containing either U0126 (30 μM) (Sigma-Aldrich), MK2206 (9 μM) (Sigma-Aldrich), U0126 (30 μM) and MK2206 (9 μM), or vehicle (DMSO) only, for 4 h for LC3A/B (an autophagy marker), or for 48 h for SA-βgal activity (a biomarker of senescent cells). For SA-βgal activity, cells were subsequently washed twice with PBS and followed by a 72-h culture in drug-free medium (2%

charcoal-stripped FBS). Immunofluorescence staining for LC3A/B (D2H10, 1:100, Cell Signaling, Danvers, MA, USA) was performed. Alexa Fluor 594 (red) goat anti-rabbit IgG conjugated antibodies (Life Technologies) were used as secondary antibodies. Fluorescence histochemical detection of SA-βgal activity was performed according to the protocol published by Debacq-Chainiaux et al.[9] Both for LC3A/B and SA-βgal activity, cell nuclei were stained with 4, 6-diamidino-2-phenylindole (DAPI) (Life Technologies). Slides were analyzed with a Leica TCS SPE confocal laser-scanning microscope (Leica Microsystems, Nanterre, France).

References

1. Matsuzaki, S. & Darcha, C. Co-operation between the AKT and ERK signaling pathways may support growth of deep endometriosis in a fibrotic microenvironment in vitro. Hum. Reprod. **30,** 1606-1616 (2015).
2. Matsuzaki, S., Canis, M., Pouly, J.L. & Darcha, C. Soft matrices inhibit cell proliferation and inactivate the fibrotic phenotype of deep endometriotic stromal cells in vitro. Hum. Reprod. **31,** 541-553 (2016).
3. Matsuzaki, S. & Darcha, C. In vitro effects of a small-molecule antagonist of the Tcf/ß-catenin complex on endometrial and endometriotic cells of patients with endometriosis. PLoS One. **8,** e61690 (2013).
4. Matsuzaki, S. & Darcha, C. Involvement of the Wnt/β-catenin signaling pathway in the cellular and molecular mechanisms of fibrosis in endometriosis. PLoS One. **8,** e76808 (2013).
5. Matsuzaki, S. & Darcha, C. Antifibrotic properties of epigallocatechin-3-gallate in endometriosis. Hum. Reprod. **29,** 1677-1687 (2014).
6. Hinz, B. et al. The myofibroblast: one function, multiple origins. Am. J. Pathol. **170,** 1807-

1816 (2007). Review.

7. Paner, G.P. et al. Diagnostic utility of antibody to smoothelin in the distinction of muscularis propria from muscularis mucosae of the urinary bladder: a potential ancillary tool in the pathologic staging of invasive urothelial carcinoma. Am. J. Surg. Pathol. **33,** 91-98 (2009).
8. Syed, S., Karadaghy, A. & Zustiak, S. Simple Polyacrylamide-based Multiwell Stiffness Assay for the Study of Stiffness-dependent Cell Responses. J. Vis. Exp. **97,** e52643 (2015).
9. Debacq-Chainiaux, F., Erusalimsky, J.D., Campisi, J. & Toussaint, O. Protocols to detect senescence-associated beta-galactosidase (SA-βgal) activity, a biomarker of senescent cells in culture and in vivo. Nature Protocols. **4,** 1798 –1806 (2009).

Supplementary Figure 1

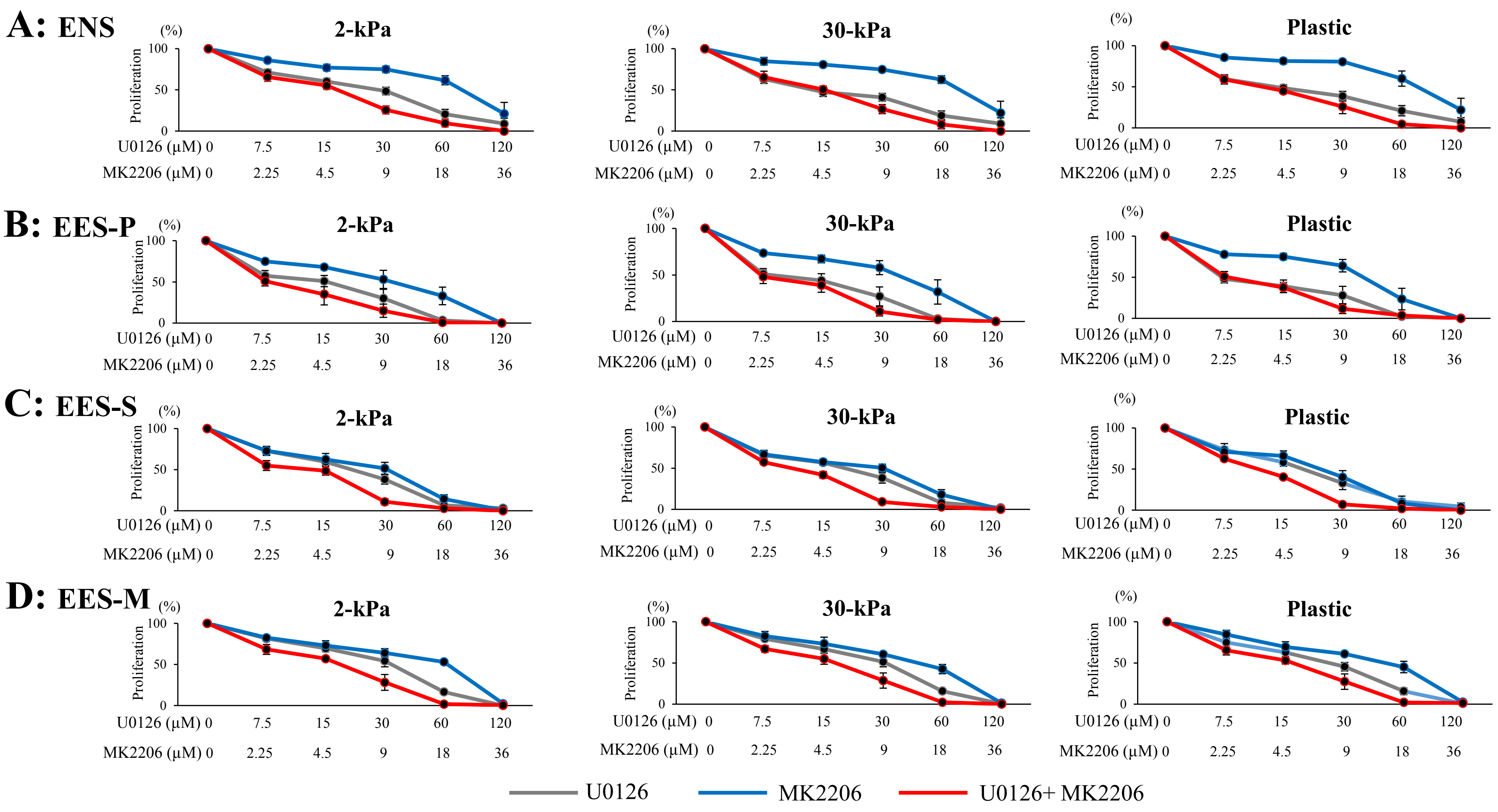

Supplementary Figure 2

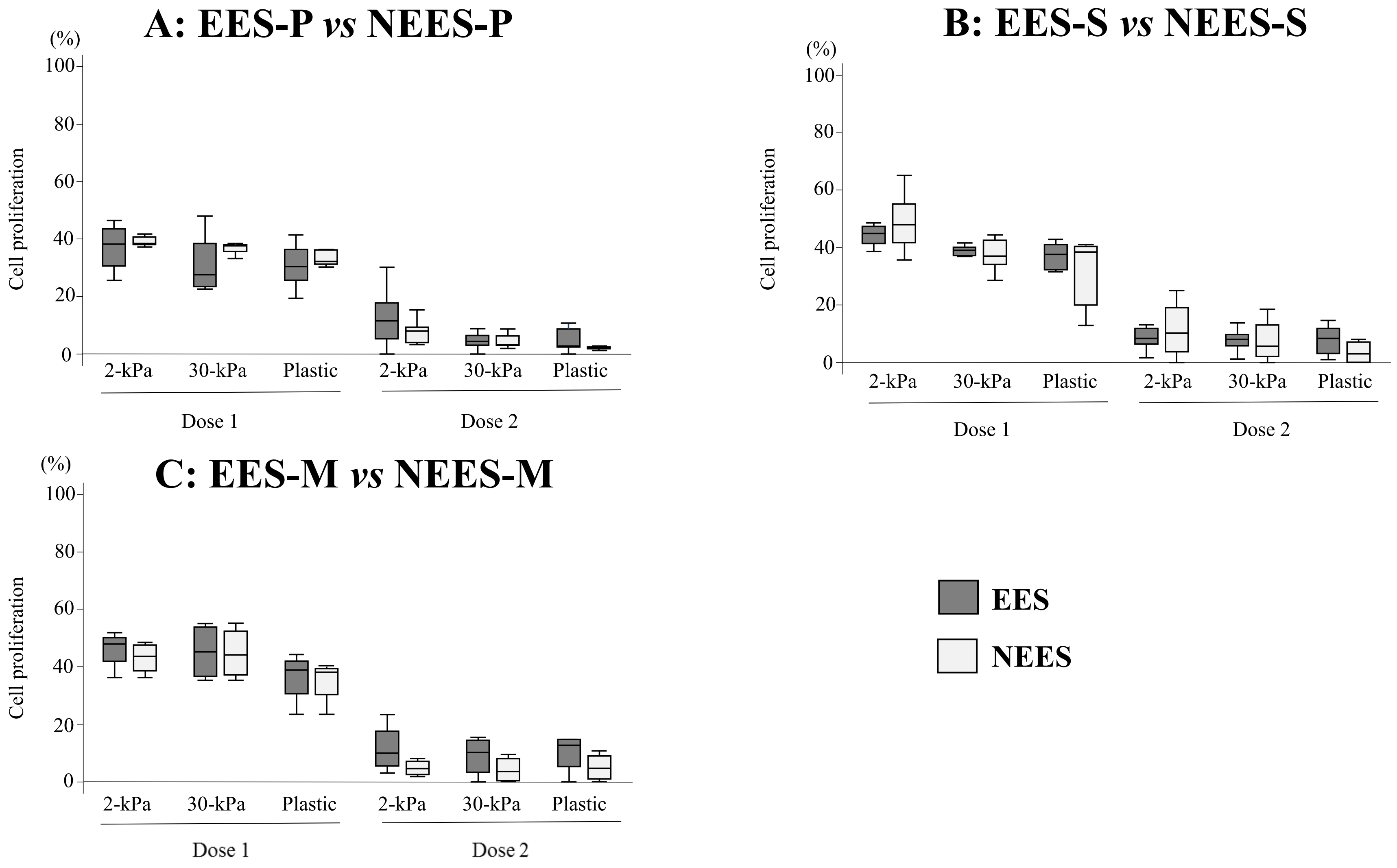

Supplementary Figure 3

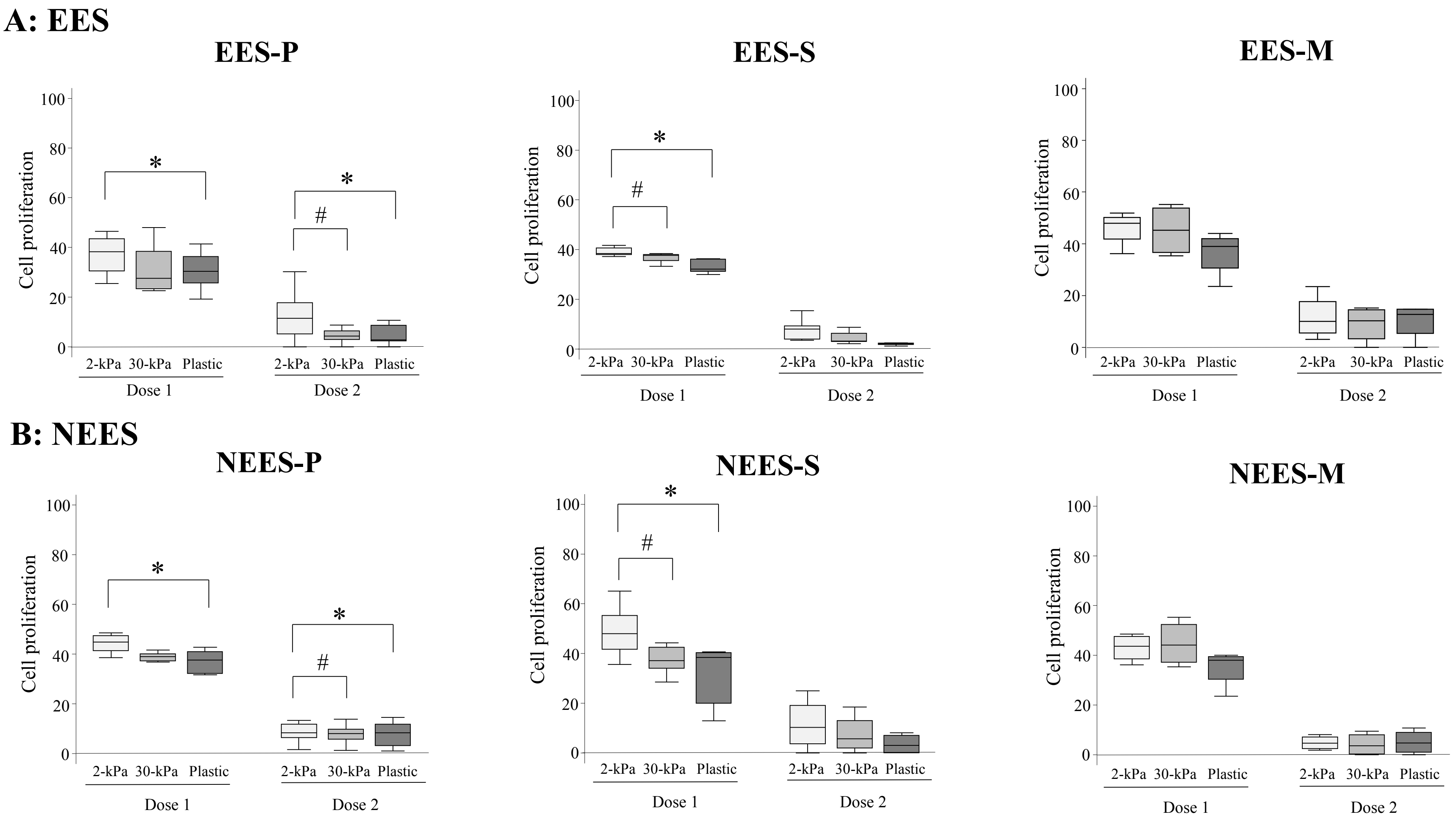

Supplementary Figure 4

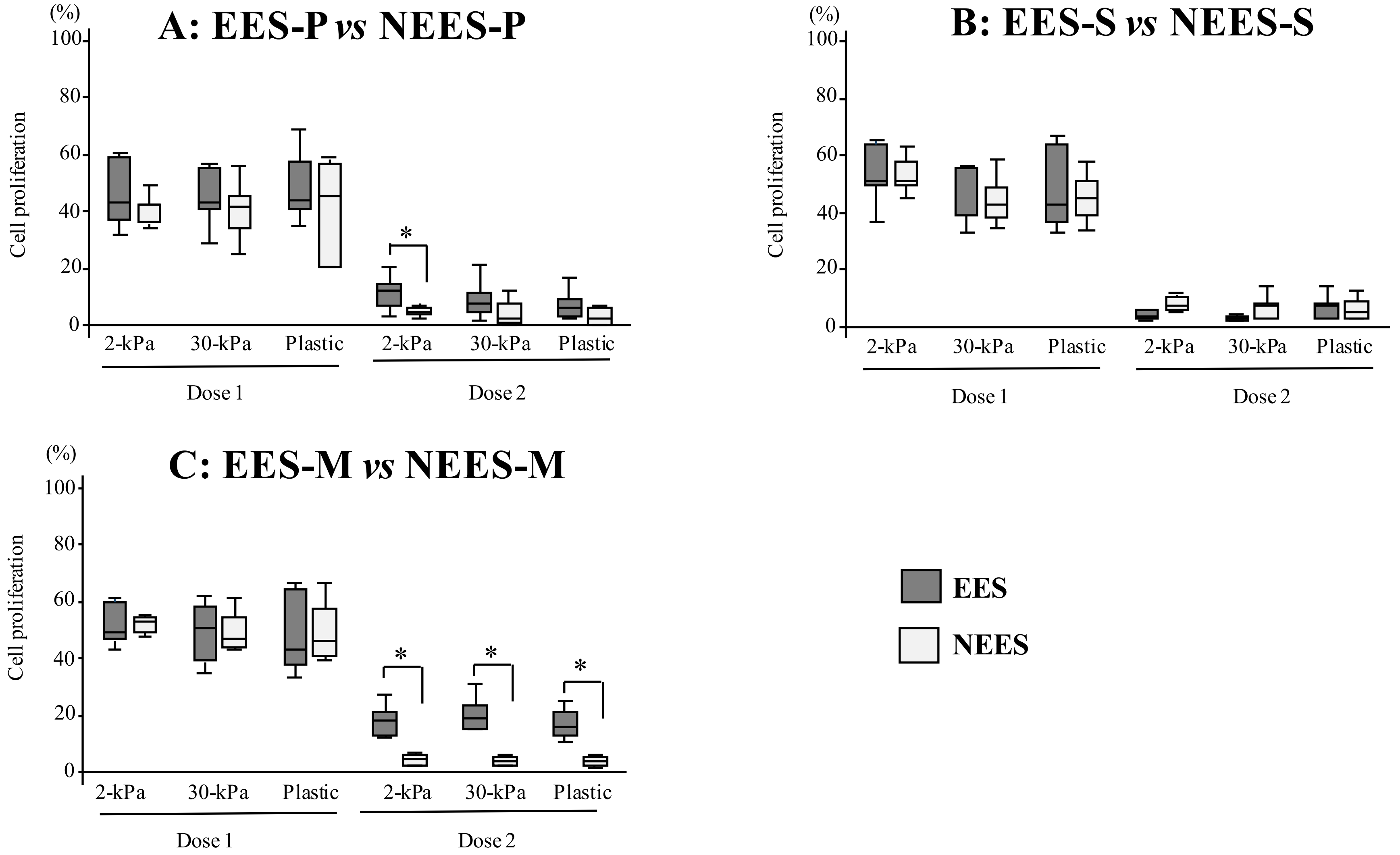

Supplementary Figure 5

A: EES

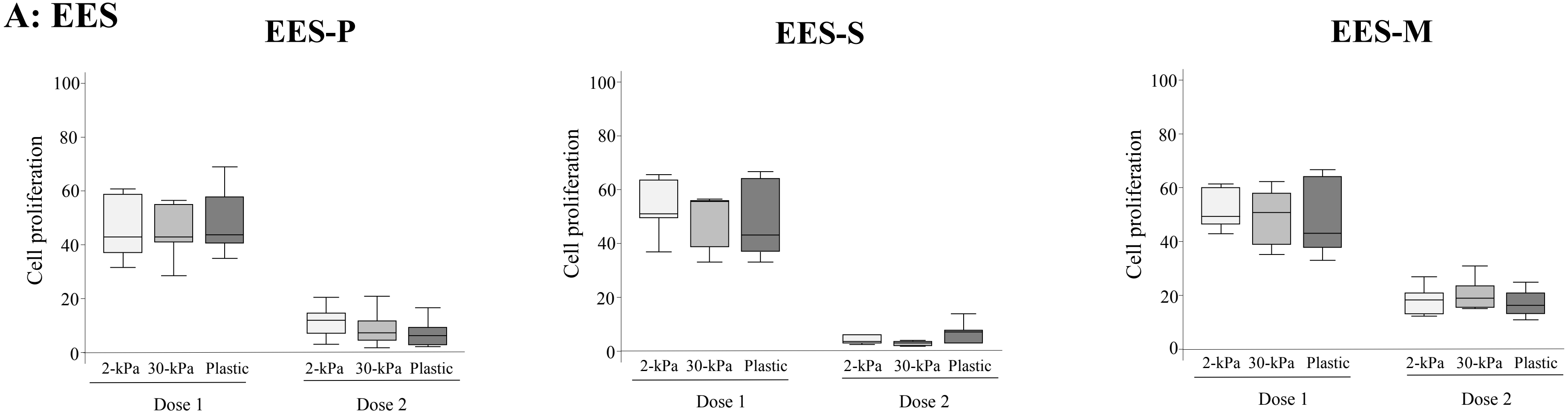

B: NEES

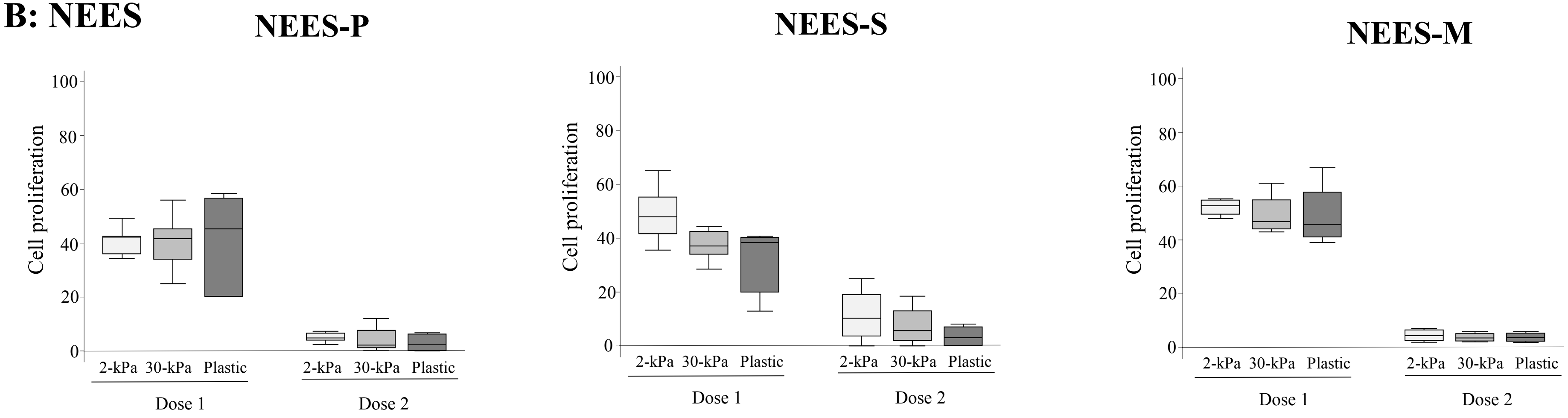

Supplementary Figure 6A

A: DES

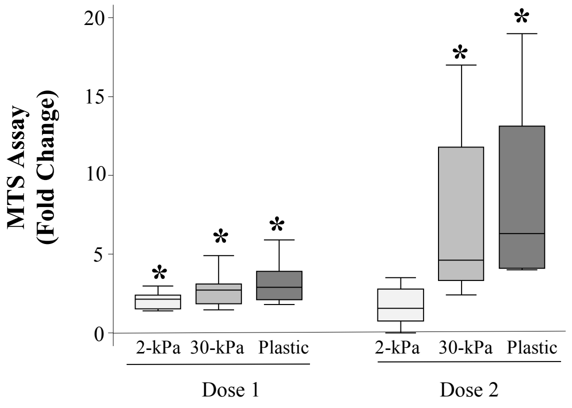

Supplementary Figure 6B & 6C

B: EES

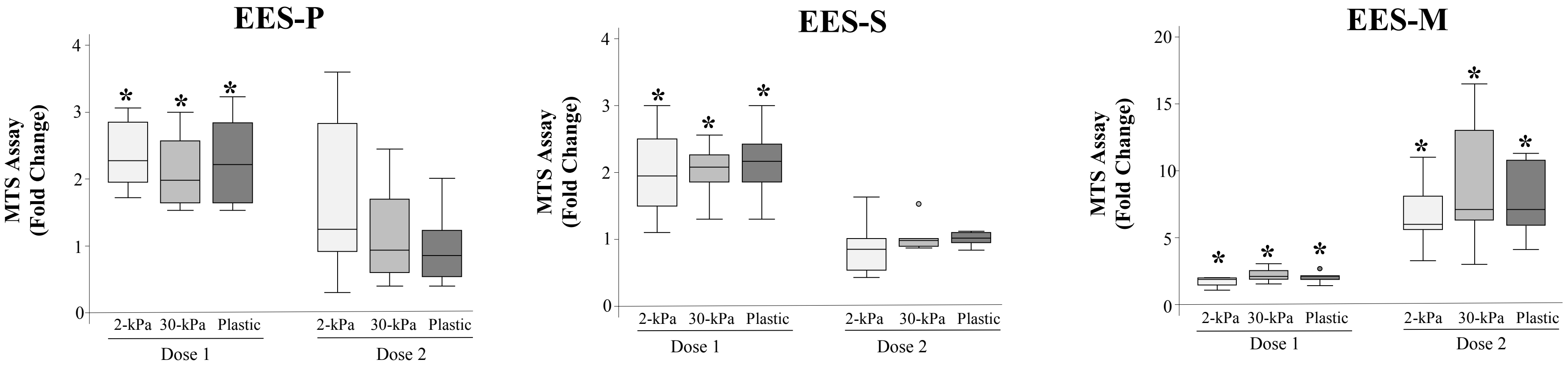

C: NEES

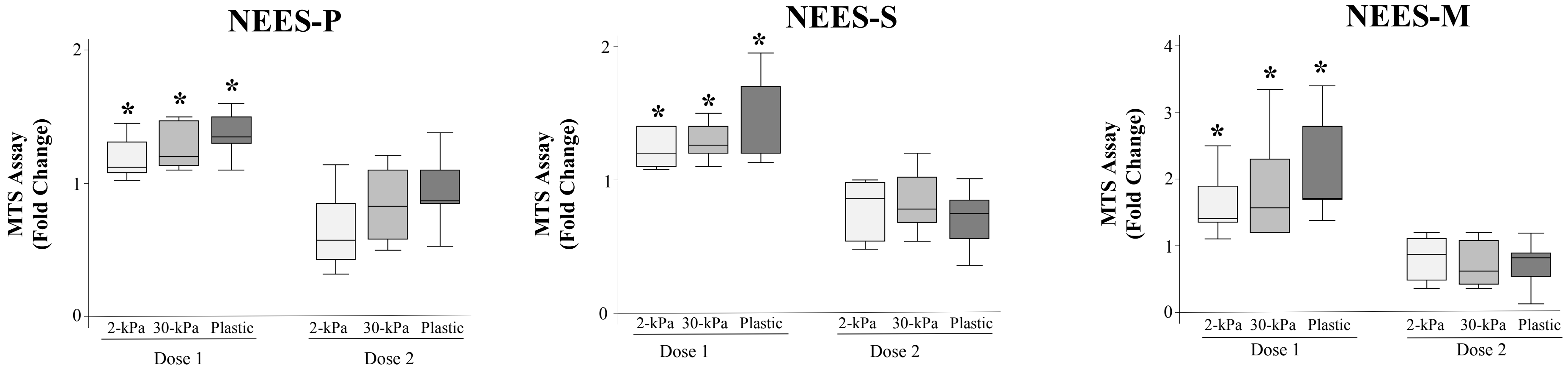

Supplementary Figure Legends

Supplementary Figure 1: Dose response curve for cell proliferation in deep endometriotic stromal cells (DES) (A) (n=10), endometrial stromal cells of patients with endometriosis (EES) derived from the proliferative phase (EES-P) (B) (n=5), EES derived from the secretory phase (EES-S) (C) (n=5), and EES derived from the menstrual phase (EES-M) (D) (n=5) treated with either U0126 alone, MK2206 alone, or combination U0126 and MK2206.

Cells were grown on polyacrylamide gel substrates (PGS) of varying stiffness (2 or 30 kPa) or plastic. Percent cell proliferation is calculated relative to vehicle-treated cells.

Supplementary Figure 2: Effects of combined treatment with U0126 and MK2206 on cell proliferation in EES versus endometrial stromal cells without endometriosis (NEES).

(A): EES-P (n=10) versus NEES-P (n=5).

(B): EES-S (n=6) versus NEES-S (n=5).

(C): EES-M (n=5) versus NEES-M (n=5).

Dose 1: U0126 (15 μM) and MK2206 (4.5 μM).

Dose 2: U0126 (30 μM) and MK2206 (9 μM).

NEES-P: NEES derived from the proliferative phase.

NEES-S: NEES derived from the secretory phase.

NEES-M: NEES derived from the secretory phase.

Numerical values are presented as box and whisker plots showing medians and the smallest and largest data points ≤1.5 × IQR from the 25th and 75th percentiles, respectively.

Supplementary Figure 3: Effects of substrates of varying stiffness (2- or 30-kPa PGS, or plastic) on inhibition of cell proliferation of EES and endometrial stromal cells without endometriosis (NEES)

(A): EES; EES-P (n=10), EES-S (n=6) and EES-M (n=5).

(B): NEES-P (n=5), NEES-S (n=5) and NEES-M (n=5).

Dose 1: U0126 (15 μM) and MK2206 (4.5 μM).

Dose 2: U0126 (30 μM) and MK2206 (9 μM).

*: p<0.05: 2-kPa PGS versus plastic.

#: p<0.05: 2-kPa PGS versus 30-kPa PGS

Numerical values are presented as box and whisker plots showing medians and the smallest and largest data points ≤1.5 × IQR from the 25th and 75th percentiles, respectively.

Supplementary Figure 4: Cell proliferation of EES versus NEES after a 72-h discontinuation of combination U0126 and MK2206.

(A): EES-P (n=10) versus NEES-P (n=5).

(B): EES-S (n=6) versus NEES-S (n=5).

(C): EES-M (n=5) versus NEES-M (n=5).

Cells were grown on PGS of varying stiffness (2 or 30 kPa) or plastic.

Dose 1: U0126 (15 μM) and MK2206 (4.5 μM).

Dose 2: U0126 (30 μM) and MK2206 (9 μM).

*: p<0.05 EES versus NEES.

Numerical values are presented as box and whisker plots showing medians and the smallest and largest data points ≤1.5 × IQR from the 25th and 75th percentiles, respectively.

Supplementary Figure 5: Effects of substrates of varying stiffness (2- or 30-kPa PGS, or plastic) on cell survival of EES and NEES

(A): EES; EES-P (n=10), EES-S (n=6) and EES-M (n=5).

(B): NEES-P (n=5), NEES-S (n=5) and NEES-M (n=5).

Dose 1: U0126 (15 μM) and MK2206 (4.5 μM).

Dose 2: U0126 (30 μM) and MK2206 (9 μM).

Numerical values are presented as box and whisker plots showing medians and the smallest and largest data points ≤1.5 × IQR from the 25th and 75th percentiles, respectively.

Supplementary Figure 6: Intra-group comparison of cell proliferation of DES (A), EES (B) and NEES (C) grown on PGS of varying stiffness (2 or 30 kPa) or plastic after a 72-h discontinuation of U0126 and MK2206.

(A): DES (n=14)

(B): EES-P (n= 10), EES-S (n=6) and EES-M (n=5)

(C): NEES-P (n=5), NEES-S (n=5) and NEES-M (n=5).

Dose 1: U0126 (15 μM) and MK2206 (4.5 μM).

Dose 2: U0126 (30 μM) and MK2206 (9 μM).

*: $p<0.05$: after a 72-h drug discontinuation versus after a 48-h treatment.

Cell proliferation after a 72-h drug discontinuation is expressed as fold change relative to that after a 48-h treatment. Numerical values are presented as box and whisker plots showing medians and the smallest and largest data points ≤1.5 × IQR from the 25th and 75th percentiles, respectively.

Supplemental Table 1: Combination index (CI) as a function of effect dose (ED) in deep endometriotic stromal cells (ENS), endometrial stromal cells of patients with endometriosis, derived from the proliferative (EES-P), the secretory (EES-S) and the menstrual (EES-M) phases.

1. CI: ENS

	2-kpa	30-kPa	Plastic
ED50	0.82 (0.47-0.89)	1.05 (0.61-1.25)	1.08 (0.775-1.43)
ED75	0.35 (0.14-0.63)	0.48 (0.19-0.84)	0.70 (0.43-0.89)
ED90	0.17 (0.05-0.48)	0.32 (0.06-0.69)	0.53 (0.2-0.69)
ED95	0.10 (0.01-0.44)	0.28 (0.03-0.58)	0.48 (0.11-0.71)

2. CI: EES-P

	2-kpa	30-kPa	Plastic
ED50	1.13 (1.00-1.20)	1.20 (1.04-1.24)	1.38 (1.08-1.5)
ED75	1.24 (1.15-1.69)	1.35 (1.20-1.36)	1.45 (1.20-1.80)
ED90	1.29 (1.26-1.55)	1.48 (1.27-1.86)	1.71 (1.53-2.3)
ED95	1.33 (1.25-1.35)	1.57 (1.4-1.85)	1.59 (1.21-2.01)

3. CI: EES-S

	2-kpa	30-kPa	Plastic
ED50	0.98 (0.91-1.05)	1.46 (1.15-1.85)	1.19 (1.02-1.77)
ED75	0.98 (0.95-1.26)	1.34 (1.21-1.75)	1.05 (0.95-1.60)
ED90	1.04 (0.94-1.21)	1.30 (1.13-1.85)	1.10 (0.96-1.47)
ED95	1.11 (0.97-1.12)	1.34 (1.05-2.0)	1.18 (0.95-1.40)

4. CI: EES-M

	2-kpa	30-kPa	Plastic
ED50	1.07 (0.98-1.46)	1.24 (1.00-1.56)	1.27 (1.12-1.70)
ED75	1.01 (1.00-1.21)	1.14 (1.01-1.41)	1.21 (1.15-1.55)
ED90	1.09 (0.94-1.20)	1.18 (1.06-1.21)	1.21 (1.04-1.41)
ED95	1.12 (0.90-1.18)	1.23 (0.994-1.24)	1.33 (1.01-1.37)

Cells were grown on polyacrylamide gel substrates (PGS) of varying stiffness (2-, or 30 kilopascal (kPa)) or plastic.

ED values of 50, 75, 90 or 95 correspond to 50, 75, 90 or 95% growth inhibition, respectively.

CI <0.9, 0.9-1.1, and >1.1 represent synergism, additivity, and antagonism, respectively.

The data is color coded with blue representing numerical values >0.9 (no synergy), yellow indicates values <0.9 (synergy).

Data are shown as median and interquartile range (IQR, 25th-75th percentile) of CI

Supplemental Table 2. Clinical characteristics of patients

	Endometriosis	Uterine fibroma	Tubal infertility
No of cases	73	10	11
Age [a]	32.0 (21-37)	32.0 (29-36)	29.0 (21-32)
Parity [a]	0 (0-1)	0 (0-1)	0 (0-1)
rASRM stage[b]			
I	25		
II	18		
III	14		
IV	16		

[a] Median (range)

[b] Revised American Society for Reproductive Medicine classification (rASRM) (American Society for Reproductive Medicine, 1997).

Supplemental Table 3: The numbers of samples of DES, EES, and/or NEES used for each experiment

	DES	EES			NEES		
		EES-P	EES-S	EES-M	NEES-P	NEES-S	NEES-M
Drug combination analysis	10	5	5	5			
Effects of the combination of U0126 and MK2206 on inhibition of cell proliferation	14	10	6	5	5	5	5
Annexin V staining	12	6	6	5			
qPCR & beta-galactosidase activity	6	6					
LC3A/B staining	3	3	3	3	3	3	3
IC_{50} for bafilomycin A1 and chloroquine	5						
Effects of bafilomycin A1 and chloroquine on inhibition of cell proliferation of DES grown on plastic	5						
Effects of chloroquine on inhibition of cell proliferation of DES grown on substrates of varying stiffness	6						

DES: deep infiltrating endometriotic stromal cells

EES-P: endometrial stromal cells of patients with endometriosis (EES) derived from the proliferative phase; EES-S: EES derived from the secretory phase; EES-M: EES derived from the menstrual phase

NEES-P: endometrial stromal cells of patients without endometriosi (NEES) derived from the proliferative phase; NEES-S: NEES derived from the secretory phase; NEES-M: NEES derived from the menstrual phase

Supplementary Table 4: Sequences of the primers used for mRNA quantitation by real-time RT-PCR

Gene	Sense primers	Antisense primers
Cyclin D1	5´-GTGGGTGTGCAAGCCAGGT-3´	5´-TTCCTGTCCTACTACCGCCT-3´
p53	5´-CCG CAG TCA GAT CCT AGC G-3´	5´-AAT CAT CCA TTG CTT GGG ACG-3´
p21	5´-TGC CGA AGT CAG TTC CTT GT-3´	5´-CAT GGG TTC TGA CGG ACA TC-3´
GAPDH	5'-TGCACCACCAACTGCTTAG-3'	5'-CTCTCGTTCACCTCGATCTTCA-3'

p21 : p21$^{WAF1/Cip1}$

GAPDH: glyceraldehyde 3-phosphate dehydrogenase
